# M_1 _muscarinic receptor for the development of auditory cortical function

**DOI:** 10.1186/1756-6606-3-29

**Published:** 2010-10-22

**Authors:** Karalee K Shideler, Jun Yan

**Affiliations:** 1 Department of Physiology and Pharmacology, Hotchkiss Brain Institute, Faculty of Medicine, University of Calgary, Calgary, Alberta., Canada T2N 4N1

## Abstract

The sensory cortex is subject to continuous remodelling during early development and throughout adulthood. This process is important for establishing normal brain function and is dependent on cholinergic modulation via muscarinic receptors. Five muscarinic receptor genes encode five unique receptor subtypes (M_1-5_). The distributions and functions of each subtype vary in central and peripheral systems. In the brain, the M_1 _receptor is most abundant in the cerebral cortex, where its immunoreactivity peaks transiently during early development. This likely signifies the importance of M_1 _receptor in the development and maintenance of normal cortical function. Several lines of study have outlined the roles of M_1 _receptors in the development and plasticity of the auditory cortex. For example, M_1_-knockout reduces experience-dependent plasticity and disrupts tonotopic mapping in the adult mouse auditory cortex. Further evidence demonstrates a role for M_1 _in neurite outgrowth and hence determining the structure of cortical neurons. The disruption of tonotopic maps in M_1_-knockout mice may be linked to alterations in thalamocortical connectivity, because the targets of thalamocortical afferents (layer IV cortical neurons) appear less mature in M_1 _knockouts. Herein we review the literature to date concerning M_1 _receptors in the auditory cortex and consider some future directions that will contribute to our understanding.

## Background

**Figure 1:**
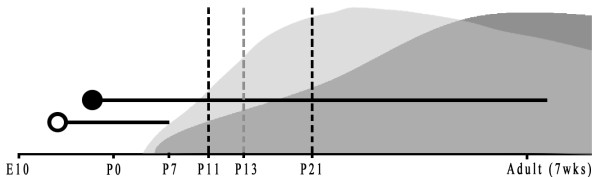
**Schematic representation of thalamocortical and cholinergic fiber ingrowth to the auditory cortex of mice**. The open circle depicits the arrival of thalamocortical afferent fibers at embryonic day (E)15-16 [1,5], and the line extending from it represents the course of their maturation to completion at postnatal day (P)7 [6]. The closed circle represents the initial invasion to the cortex by cholinergic fibers on E18-19, and the associated line, their maturation through the first 7 weeks of life [7,81]. The dotted lines delineate the boundaries of proposed critical periods of rapid reformation and maturation beginning at P11 [13] and extending to P21 [32], with the grey dotted line marking the end of an purposed early critical period at P13 [13]. The light-grey cloud represents the trends in M_1 _expression, with a peak represents the maximal adult expression levels at ~P26 and a subsequent gradual decline throughout adult life (conservative average of data from from several articles [32,38,82]). The dark-grey shaded area area represents choline acetyltransferase (ChAT) expression, again with the peak of this curve being maximal expression, rising more slowly and likely sustained longer into adult life than that for M_1 _(based on data published by Hohmann 1985 [81]).

Sensory cortices begin to form and make intracortical and subcortical connections early in the developmental regime, and the auditory cortex is no exception. The cortical plate in mice is present as early as embryonic day (E) 11 [1], at about the same time as cochlear hair cells are forming [2,3]. During early development several key neuronal projections make connections in the cortex, and here we focus on two: one consists of glutamatergic axons from thalamic relay cells, through which the cortex receives the majority of its environmental input [4], and the other comprises cholinergic axons, primarily from the basal forebrain. Before birth, at E15-16 [1,5], projections from the medial geniculate body (MGB) of the thalamus reach the cortical plate, where they first form functional connections with transitory cortical subplate neurons [1,6]. Axons of mature MGB neurons connect onto cortical pyramidal cells at or before postnatal day (P)7 [6]. Axons of cholinergic neurons similarly arrive in the cortex at E18-19 and mature during the first two months after birth [7], as shown in Figure 1. Despite the elaborate choreography of prenatal development, in rodents functional hearing develops only postnatally, as connections between hair cells and the auditory nerve become functional at P5 [6] and the ear canal opens at P9 [6,8].

Interestingly, some of the tonotopic-like organization of the auditory system is present before the sensory input of sound is available [9-11]. For example, at E15.5 the projections of spiral ganglion cells onto the cochlear nucleus are tonotopically correct, i.e., spiral ganglion neurons that will innervate the cochlear base or apex, already innervate the dorso-medial or ventro-lateral cochlear nucleus, respectively [12]. Within the cortex, tonotopic-like maps depend on spontaneous activity initially, before opening of the ear canal, and then become mature after a so-called 'critical period' of early development [13]. Mature maps are generally composed of neurons in orderly array, tuned to various frequencies appropriate to the hearing range of a given species, and they have sharp frequency-tuning curves. The generation of mature maps is highly dependent on early experience of the acoustic environment [14].

Cholinergic neurons within the nucleus basalis and the septal diagonal band complex provide the major source of cholinergic innervation of the cerebral cortex and hippocampus and play key roles in memory [15] and attention [16]. A role for acetylcholine (ACh) as a classical neuromodulator in the central nervous system - including the cerebral cortex - is supported by a wealth of published data. Despite the abundance of articles exploring the possible roles of cholinergic input, many mechanisms underlying cholinergic modulation are still not well understood. It is intriguing to consider that early neonatal ablation of cholinergic projections from basal forebrain to cortex results in considerable structural abnormalities within the cortex, such as: smaller soma size and shorter apical and basal dendritic branches of pyramidal neurons; unclear boundaries between supragranular layers; abnormal pattern-formation in layer IV; and irregular intracortical connectivity and altered distribution of thalamocortical projections in the neocortex [17-19]. The specific actions by which ACh can mediate these morphological changes is unclear. The authors of the aforementioned ablation studies hypothesized, and the evidence presented later in this review suggests, that muscarinic acetylcholine receptor (mAChR) subtype 1 (M_1_) is a key player in determining normal cortical development and function. This review will focus specifically on M_1 _and the auditory cortex, as significant recent work has been conducted in the auditory system.

Postsynaptic neurons can respond to ACh by means of a wide variety of plasma-membrane receptors, which can be divided into two major classes, nicotinic acetylcholine receptors (nAChRs) and muscarinic receptors (mAChRs) [20]. While nAChRs are ionotropic ACh-gated cation channels [20-22], mAChRs are metabotropic members of the G protein-coupled receptor superfamily [20,23,24]. At present, five mAChR genes (*m*_*1*_*-m*_*5*_) are known, which encode receptors M_1_-M_5_, respectively. All mAChR subtypes act via activation of G-proteins to influence membrane properties via different second messengers; M_1_, M_3 _and M_5 _receptors are associated with G-proteins (Gq/11), which activate phospholipase C, whereas M_2 _and M_4 _receptors are associated with G-proteins (Gi/Go), which inhibit adenylyl cyclase. A classification of some interesting functions of acetylcholine receptors is presented in Table 1.

**Table 1:** Acetylcholine Receptors and Function in Brain.

Nicotinic	Muscarinic				
	
	M_1_	M_2_	M_3_	M_4_	M_5_
Facilitates thalamocortical transmission[25]	Neurite outgrowth[26]				
Unclear roles in plasticity and development	Promotes Cell Survival[27]		Promotes Cell Survival[27]		Promotes Cell Survival[27]
	Impact auditory plasticity[28-30]	Impact auditory plasticity[30]	Impact auditory plasticity[30]	Impact auditory plasticity[30]	Impact auditory plasticity[30]
	Proposed importance in cortical development[28,29,31]				

Perinatally, both nAChRs and mAChRs are present in the mammalian cerebral cortex [31]. However, during the course of cortical development the expression (mRNA) levels of most nAChRs are constant, whereas those of mAChRs vary, having peak periods within the first several weeks of postnatal life that correspond with times of morphogenesis and synaptogenesis [32]. These patterns suggest that the presence of mAChRs could play an important role in development and maturation in the auditory cortex. On the other hand, nAChRs also undoubtedly play some role in normal cortical developmental. For example, the cortical pyramidal cells of nAChR-β2 subunit knockout mice had shorter dendritic arbours and lower spine densities than those of wild-type mice [33]. Furthermore, within the auditory system, activation of cortical nAChRs was found to enhance cognitive function [34-36]; rats that performed well in an auditory-cued active avoidance task also responded to nicotine administration with enhanced response to stimuli, as long as the tone used was close to the conditioning tone. Interestingly, in addition, nicotine administration not only enhanced the response to a closely matched tone, but it also reduced responses to spectrally distant stimuli. Because these effects were observed only in the population of rats that performed well in the initial trials, perhaps the role of nAChRs was to exert some refining effect upon plasticity [36]. In general, studying nAChR can be complex, as many combinations of different nAChR subunits can form homo- and heteromeric pentameric channels, producing a large variety of channels having varying kinetics and pharmacology. The complexity of roles of nAChR receptors in cortical development warrants a dedicated discussion, which is outside of the scope of this review.

Although the types and functional mechanisms of mAChRs are considerably less complex than those of nAChRs. a comprehensive understanding still poses serious challenges. Many organs express more than one mAChR subtype [20,37-39]. In the adult brain, all mAChR subtypes are present, and they are expressed in regionally specific patterns. The distributions and concentrations of mAChR subtypes are commonly determined by localizing mAChR mRNA with *in situ *hybridization, by identifying mAChR protein immunohistochemically, and by measuring mRNA or localizing muscarinic ligand-binding. Early experiments indicated that M_1_, M_2 _and M_4 _mRNA and protein were the most abundant mAChRs in the brain [38,39], and that M_1 _was the predominant mAChR in the cerebral cortex [32,38]. It is noteworthy that the expression of M_1 _protein in the cerebral cortex shows a specific pattern and that the patterns of immunoreactivity of M_1 _and M_2 _in the different cortical layers seem to be strongly complementary throughout development [32]. In mice, immunoreactive M_1 _protein can be detected in the brain as early as P5; and by P14, M_1_-immunoreactivity is most apparent in layer IV, whereas M_2_-immunoreactivity is strongest in layers II/III and V [32]. These results suggest that M_1 _and M_2 _could be important to the differentiation of unique phenotypes of cortical neurons during early development [32].

A plethora of intrinsic and extrinsic cues guide cell migration and other events in the development of the cerebral cortex. Some key players in cortical formation include the proteins reelin, which controls migration and positioning of cortical neurons[40,41]; cyclin-dependent kinase 5 (Cdk5), which is part of the reelin signaling cascade and also aids in appropriate migration[41,42]; and the Slit family of proteins, acting through Robo receptors, which may guide cortical interneurons into the cortex and play roles in dendritic growth and axonal pathfinding [43,44]. Additionally, various microtubule proteins [41], centrosome proteins [41] and neurotrophic factors [45] also support normal development, maturation and function. Data suggest that cholinergic neuronal activity, involving specifically M_1 _mAChR, regulates the expression of neurotrophins such as brain-derived neurotrophic factor (BDNF) and nerve growth factor (NGF) [46-48]. Other links between mAChRs and important regulators of development seem likely but have not yet been established. Given the paucity of current literature, the role(s) of mAChRs in developmental programming - especially within the auditory system - would appear to be an area ripe for further investigation.

In addition to the data discussed above, the results of a few experiments have more directly demonstrated a role of mAChRs and M_1 _in determining the structure of neurons. Transfection of several cell lines with cDNA for rat choline acetyltransferase (ChAT) stimulated neurite outgrowth and produced a more differentiated phenotype [49]. Further investigation demonstrated that mAChRs were present on these cells, leading to the hypothesis that they were essential to the neurite outgrowth [50], and the activation of mAChRs was shown to be sufficient to initiate neurite outgrowth and the induction of synapsin 1 [49,50]. Synapsin 1, a synaptic vesicle-associated protein that is expressed during synaptogenesis, is involved classically in vesicle release; but it also promotes the elongation of neurites and mediates the formation of reciprocal contacts between neurites and the functional maturation of synapses [51,52]. Activation of mAChR by pretreatment with oxotremorine-M, a selective mAChR agonist, has also been shown to promote cell survival [27,53]; this could be important during neuronal development. Furthermore, M_1 _is involved in signaling pathways that are active during synaptogenesis, such as activation of the gamma (γ) and epsilon (ε) isoforms of protein kinase C (PKC) [26,31] and the mitogen-activated protein kinase (MAPK) pathway [31,54]. *In vitro *experiments have demonstrated that specifically M_1 _activation can signal through PKCε, resulting in robust outgrowth of neurites from pyramidal cells [26]. These *in vitro *studies suggest an interpretation that M_1 _contributes to neurite outgrowth *in vivo *as well; the length of dendrites of layer 4 multipolar granular cells in the auditory cortex was significantly shorter in M_1 _knockout mice than in wild-type controls [28]. Evidence from ablation of cholinergic inputs also suggests that M_1 _signalling is essential for the morphological maturation of the cerebral cortex [31]. While direct or indirect signalling events that occur after M_1 _activation may help to regulate the morphological parameters of cortical neurons, specifically, the length of their neurites, certainly many other factors could contribute to the regulation of neurite outgrowth. Moreover, in the auditory cortex, the dendrites of layer 4 neurons were significantly shorter in M_1 _knockout mice, whereas those of layer 5 neurons were not significantly different from wild-type controls [28]. Very few morphological findings have been reported in investigations of mAChR knockout mice. Whether or not stunted neurite outgrowth is observed in other cortical areas is currently unknown. It is possible that M1 plays some previously unexplored role in layer 4 of the auditory cortex; conversely, the shortening of dendrites in the absence of M1signalling *in vivo *may be a more general phenomenon than has been documented so far. This observation may be indicative of an alteration in either the thalamic innervation of the cortex, or the formation of synapses from thalamocortical afferents onto cortical neurons (usually in layer 4), in mice lacking M_1 _receptors [28].

A regulatory role for mAChRs, especially M_1_, has also been confirmed in development and plasticity in other areas of sensory cortex. In the visual system, intracortical infusion of pirenzepine, a moderately selective M_1_-receptor antagonist, significantly reduces ocular dominance plasticity due to postnatal monocular deprivation in cats [55]. In the somatosensory system, it has been demonstrated the Ca^2+^-currents generated by mAChR-activation help to synchronize neuronal responses [56]. Synchronous firing, early in the development of networks such as the thalamocortical connections, is believed to promote the normal development of these cortical structures [56]. Lastly, in studies investigating learning-induced auditory plasticity, cortical application of the mAChR antagonists atropine or scopolamine significantly decreased the frequency-specific plasticity of the auditory cortex when evoked by auditory fear-conditioning [57-59] or basal forebrain stimulation paired with a sound [30,60-62].

More specifically, our laboratory has previously demonstrated that the auditory systems of 4-week-old M_1 _knockout mice are physiologically abnormal in several ways. The reported deficits include, firstly, a reduction in number of cortical neurons tuned to 20 kHz and above; this may reflect some form of altered maturation, because after ear-opening, the best frequency of neurons tends to shift toward higher frequencies [28]. It was also reported that, in M_1 _knockout mice, greater numbers of neurons in the auditory cortex had multi-peaked frequency-tuning curves [29]; in contrast, fewer than half of the neurons in the primary auditory cortex and anterior auditory cortex of wild type mice had multi-peaked best frequencies [28]. Although the exact cause of more multi-peaked tuning curves is unknown, it is reasonable to suggest that an abnormality in thalamocortical input is responsible. This proposed abnormality could be due to a minor misguiding of thalamocortical fibres, or perhaps a network-mediated effect in which there is a lack of inhibition of a response to other tones [63]. Also likely related to this is the observation that the tonotopic maps in auditory cortex of M_1 _knockout mice are disrupted. Typically, auditory tonotopy is characterized by a systematic organization of neural responses, in which neurons in the dorsal portion of the auditory cortex are tuned to high frequencies, whereas those in rostral and caudal portions are tuned to lower frequencies [28]. In M_1 _knockout mice, however, this systematic dorsal to rostro-caudal pattern was not clear, and it was difficult to delineate the subdivisions of the auditory cortex on the basis of tonotopic organization [28]. Both the lack of cells with a best frequency over 20 kHz, and the poor organization of the tonotopic map of the primary and anterior auditory cortex, suggest that a lack of M_1 _may stunt functional maturation of the cortex and interfere with developmental plasticity [28]. Moreover, it has been demonstrated that ACh release within the auditory cortex activates particularly the M_1 _receptor, thereby enhancing postsynaptic activity and glutamate-mediated membrane potentiation via AMPA- (α-amino-3-hydroxyl-5-methyl-4-isoxazole-propionate) and NMDA- (N-methyl-D-aspartic acid) type ionotropic glutamate receptors [64-66]. It has been shown that blockade of AMPA- and NMDA-receptors by both APV ((2R)-amino-5-phosphonopentanoate) and CNQX (6-cyano-7-nitroquinoxaline-2,3-dione) reduces the length and density of postsynaptic dendrites [67]. It would be interesting to investigate whether antagonizing AMPA- and NMDA-receptors, via long-term continuous neonatal microinjection to the auditory cortex, would produce the same tonotopic disorganization and morphological findings as M1 knockout. Conversely, an AMPA- or NMDA-receptor-mediated attempt at rescuing the M_1 _knockout could also be informative for determining a mechanism by which M_1 _acts in the auditory cortex. The result of deletion the *m*_1 _gene seems to be an auditory cortex that is both functionally and structurally less mature than wild-type, and in which thalamocortical connectivity has been altered.

In regards to plastic changes, frequency-specific plasticity was smaller in magnitude, and shifted less towards the presented tone and for a shorter duration, in M_1_-deficient mice [29]. This suggests that M_1 _-receptors are critical for input-specific plasticity in the auditory cortex and may influence learning-induced or experience-dependent cortical plasticity [29]. Behavioural studies demonstrate that M_1 _mutants do well in matching-to-sample problems, but are deficient in solving non-matching-to-sample working memory tasks [68]. On the other hand, some authors have reported that M_1 _receptors play an important role in the regulation of locomotor activity and have described the mutants as having a hyperactivity phenotype, suggesting that this - rather than any cognitive deficit - may be responsible for their impaired performance in a behavioural test [69]. While increased locomotor activity does nothing to explain the decreased input-specific plasticity in the auditory cortex of M_1 _knockout mice; it does serve to demonstrate the complexity of the issue at hand. M_1 _does seem to impact some part of learning, memory and plasticity; however, the mechanism or mechanisms underlying its influence remain elusive. Investigation of conditional knockout mice could be a useful technique, not only for the elimination of potentially extraneous effects in other organ systems (complicating the data), but also for pinpointing important windows of time during which M_1 _mediates critical developmental processes. Also, aiming conditional knockout techniques at thalamocortical neurons or hippocampal neurons, for example, could clarify whether M_1 _acts differently in different cell populations and help to delineate its role in learning and memory.

Finally, it would seem prudent to discuss pathological conditions in which M_1 _may be involved. Although mAChRs have been implicated in numerous pathologies, the precise nature of their involvement is frequently controversial. Examples include: schizophrenia [70-74], Alzheimer disease [75-77] and other dementias [77,78]. Schizophrenia may be an interesting focus for studying the role of M_1 _in the auditory system, as auditory hallucinations are a hallmark of the disorder [74]. Clinical trials of muscarinic drugs in these disorders have met with mixed success. Psychiatric illnesses that affect children may also be interesting, and the possible roles of M_1 _in the pathogenesis of (e.g.) autism [79] and attention-deficiency disorder [80] are also being investigated. Certainly, as our basic understanding of the roles of these receptors increases, it is likely that our insight into the associated pathologies will increase also.

## Conclusion

In summary, a growing body of literature suggests that M_1 _is important to the normal development and physiology of the auditory cortex. While many questions remain about the precise mechanisms of action *in vivo*, several *in vitro *studies highlight specific signaling pathways, involving molecular regulators such as PKC [26] and NMDA-receptors [64-66]. Although some studies have pointed towards possible mechanisms, most of the available data consist only of correlations that suggest an involvement of M_1 _in development of auditory cortex and thalamocortical projections but do not demonstrate causal roles. Further investigation of the auditory system of M_1 _knockout mice may lead to a better understanding of the role of a single subtype of mAChR, uncomplicated by the limited specificity of muscarinic drugs [21]. However, since M_1 _is expressed in several organs and may play different roles in the embryo, neonate and adult, investigation of conditional knockouts is likely to be much more useful. The direct role of M_1 _in thalamocortical connectivity in the cortex is of particular interest, given the critical importance of thalamic input for sensory processing and auditory perception in the cortex.

## Competing interests

The authors declare that they have no competing interests.

## Authors' contributions

KS searched the literature and drafted the manuscript. JY conceived of the topic of the review. All authors read and approved the final manuscript.
